# Onasemnogene abeparvovec for presymptomatic infants with three copies of *SMN2* at risk for spinal muscular atrophy: the Phase III SPR1NT trial

**DOI:** 10.1038/s41591-022-01867-3

**Published:** 2022-06-17

**Authors:** Kevin A. Strauss, Michelle A. Farrar, Francesco Muntoni, Kayoko Saito, Jerry R. Mendell, Laurent Servais, Hugh J. McMillan, Richard S. Finkel, Kathryn J. Swoboda, Jennifer M. Kwon, Craig M. Zaidman, Claudia A. Chiriboga, Susan T. Iannaccone, Jena M. Krueger, Julie A. Parsons, Perry B. Shieh, Sarah Kavanagh, Melissa Wigderson, Sitra Tauscher-Wisniewski, Bryan E. McGill, Thomas A. Macek

**Affiliations:** 1 Clinic for Special Children, Strasburg, PA USA; 2 Penn Medicine-Lancaster General Hospital, Lancaster, PA USA; 3 Departments of Pediatrics and Molecular, Cell & Cancer Biology, University of Massachusetts School of Medicine, Worcester, MA USA; 4 Department of Neurology, Sydney Children’s Hospital Network, Sydney, NSW Australia; 5 School of Clinical Medicine, UNSW Medicine and Health, UNSW Sydney, Sydney, NSW Australia; 6 The Dubowitz Neuromuscular Centre, University College London, Great Ormond Street Institute of Child Health & Great Ormond Street Hospital, London, UK; 7 National Institute of Health Research, Great Ormond Street Hospital Biomedical Research Centre, London, UK; 8 Institute of Medical Genetics, Tokyo Women’s Medical University, Tokyo, Japan; 9 Center for Gene Therapy, Nationwide Children’s Hospital, Columbus, OH USA; 10 Department of Pediatrics and Department of Neurology, The Ohio State University, Columbus, OH USA; 11 Department of Paediatrics, MDUK Oxford Neuromuscular Centre, Oxford, UK; 12 Neuromuscular Reference Center, Department of Pediatrics, CHU & University of Liège, Liège, Belgium; 13 Department of Pediatrics, Neurology & Neurosurgery, Montreal Children’s Hospital, McGill University, Montreal, QC Canada; 14 Department of Pediatrics, Nemours Children’s Hospital, Orlando, FL USA; 15 Center for Experimental Neurotherapeutics, St. Jude Children’s Research Hospital, Memphis, TN USA; 16 Department of Neurology, Massachusetts General Hospital, Boston, MA USA; 17 Department of Neurology, University of Wisconsin School of Medicine and Public Health, Madison, WI USA; 18 Washington University School of Medicine, St. Louis, MO USA; 19 Division of Pediatric Neurology, Columbia University Medical Center, New York, NY USA; 20 Department of Pediatrics, University of Texas Southwestern Medical Center, Dallas, TX USA; 21 Department of Neurology, Helen DeVos Children’s Hospital, Grand Rapids, MI USA; 22 Department of Pediatrics, University of Colorado School of Medicine, Aurora, CO USA; 23 Department of Neurology, David Geffen School of Medicine at UCLA, Los Angeles, CA USA; 24 Novartis Gene Therapies, Inc., Bannockburn, IL USA; 25 Translational Medicine, Novartis Institutes for BioMedical Research, Cambridge, MA USA

**Keywords:** Gene therapy, Development

## Abstract

Most children with biallelic *SMN1* deletions and three *SMN2* copies develop spinal muscular atrophy (SMA) type 2. SPR1NT (NCT03505099), a Phase III, multicenter, single-arm trial, investigated the efficacy and safety of onasemnogene abeparvovec for presymptomatic children with biallelic *SMN1* mutations treated within six postnatal weeks. Of 15 children with three *SMN2* copies treated before symptom onset, all stood independently before 24 months (*P* < 0.0001; 14 within normal developmental window), and 14 walked independently (*P* < 0.0001; 11 within normal developmental window). All survived without permanent ventilation at 14 months; ten (67%) maintained body weight (≥3rd WHO percentile) without feeding support through 24 months; and none required nutritional or respiratory support. No serious adverse events were considered treatment-related by the investigator. Onasemnogene abeparvovec was effective and well-tolerated for presymptomatic infants at risk of SMA type 2, underscoring the urgency of early identification and intervention.

## Abstract (web summary)

For infants with three copies of *SMN1* at risk for spinal muscular atrophy (SMA) type 1, onasemnogene abeparvovec improves ventilator-free survival and nutritional/respiratory independence and allows motor development indistinguishable from healthy children without SMA.

## Main

Spinal muscular atrophy (SMA) is an autosomal recessive neuromuscular disease caused by deficiency of survival motor neuron (SMN) protein resulting from biallelic deletions or pathogenic variants of the *SMN1* (*survival motor neuron 1*) gene. SMN protein is essential for the development and survival of motor neurons in the ventral spinal cord^1^. *SMN2*, a homologous gene to *SMN1*, partially compensates for *SMN1* loss by producing low amounts of SMN protein^2^. *SMN2* copy number correlates with SMA onset and severity. Patients with three copies of *SMN2* may develop SMA types 1, 2 or 3, but the presence of three copies is 54% predictive of intermediate-severity SMA type 2 with onset between 7 months and 18 months of age, 15% predictive of type 1 and 31% predictive of the milder type 3 phenotype^3^.

Untreated children with SMA type 2 experience relatively rapid neuromuscular decline before 13 years of age, followed by more gradual debilitation through adulthood^4,5^. Patients with SMA type 2 achieve the ability to sit independently, but few stand and none walk independently. With advancing age, nearly all patients with SMA type 2 develop dysphagia, joint contractures, scoliosis, and restrictive lung disease, and some may lose the ability to sit independently^6–9^. SMA type 3 causes less severe disability than SMA type 2, with patients being able to stand and walk independently, although with increasing difficulty as they age. Patients with SMA type 3 have later symptom onset and develop fewer and less severe musculoskeletal, respiratory, and feeding problems^7,8,10–12^. Considerable heterogeneity within this clinical framework exists^10,11^. Observational studies of SMA type 2 and type 3 use continuous variables such as measures of motor performance, upper limb strength and activity, pulmonary function, and compound motor action potential (CMAP)^7,8,12–15^. However, the slow pace of clinical deterioration can obscure changes in these measures over intervals typical of clinical trials^8,12^, and periods of 24 months or longer may be needed to assess the natural progression of SMA type 2 or type 3.

Two approved therapies for SMA (nusinersen and risdiplam) increase SMN protein production via modified splicing of *SMN2* and require serial dosing^16^. A third approved disease-modifying treatment, onasemnogene abeparvovec, is a gene replacement therapy that delivers *SMN* cDNA using an adeno-associated virus-9 (AAV9) vector designed for one-time intravenous infusion^17^. Because of early successes with SMA treatments, the United States and several other countries have implemented widespread neonatal screening for *SMN1* deletions, enabling identification of children at risk for SMA before symptom onset. This has critical implications for therapeutic interventions^18–20^.

Single-center case series^21–23^, an observational cohort study^24^, post-marketing data^25,26^ and the RESTORE patient registry^27–29^ demonstrate the safety and efficacy of onasemnogene abeparvovec for symptomatic patients with SMA with three *SMN2* copies. However, few interventional studies have targeted children with three *SMN2* copies who are at risk for SMA but have yet to demonstrate signs of disease. This group is largely underrepresented in clinical trials.

Results from a Phase II study of nusinersen (NURTURE) indicate the potential of disease-modifying therapy for presymptomatic children at risk for SMA type 2. Ten children with three copies of *SMN2* started nusinersen between 3 days and 42 days of age, before symptom onset^30^. All children achieved independent sitting and walking (most within the World Health Organization (WHO) normal reference interval), and none required respiratory intervention.

SPR1NT was the first Phase III study of onasemnogene abeparvovec for the treatment of presymptomatic infants at risk for SMA types 1, 2 or 3. The SPR1NT trial focused on efficacy measures, such as motor milestones, as they compared with normal developmental benchmarks^31^ and the ability to survive and thrive without mechanical interventions. We also compared efficacy and exploratory measures with the Pediatric Neuromuscular Clinical Research (PNCR) natural history population, which enrolled infants with SMA and two or three copies of *SMN2*^14^. A total of 29 SPR1NT participants comprised 14 children with two copies of *SMN2* and 15 with three copies of *SMN2*. The former cohort is the subject of a companion paper^32^. Here we focus on the 15 SPR1NT participants with three copies of *SMN2* (hereafter referred to as the three-copy cohort) and provide important new efficacy and safety data about neonatal AAV9 vector infusion in this population. Safety and efficacy data from both cohorts have critical implications for newborn screening programs and the clinical timing of therapeutic intervention.

## Results

### Screening and demographics

Of 44 newborns screened for SPR1NT who had biallelic *SMN1* deletions and two or three copies of *SMN2*, 14 were excluded. The most common reasons for exclusion were clinical signs at screening or immediately before dosing that were, in the opinion of the investigator, strongly suggestive of SMA (*n* = 4) and peroneal nerve to tibialis anterior CMAP <2 mV (*n* = 4) (Supplementary Table 1 and Supplementary Fig. 1). A total of 15 infants (female, *n* = 9; 60%) with three *SMN2* copies were enrolled in SPR1NT. Children in the three-copy cohort were born between 35 and 41 gestational weeks (median, 39.0) at a median weight of 3.4 kg (range, 2.55–3.81) (Table 1). Ten children were born at a gestational age less than 40 weeks, and one was born at a gestational age less than 37 weeks. None of the 15 infants had a c.859 G>C modifier variant, which is associated with a milder disease course. Most children (*n* = 13; 87%) were diagnosed by newborn screening. The 14 infants diagnosed after birth had a confirmed molecular diagnosis at a median age of 8.0 days (range, 2–26).

**Table 1:** Baseline characteristics of children with three copies of *SMN2*

Baseline characteristics	All patients (*n* = 15)^a^
Age at dosing, days^b^	
Mean (s.d.)	28.7 (11.68)
Median (range)	32.0 (9–43)
Gestational age at birth, weeks	
Mean (s.d.)	38.8 (1.47)
Median (range)	39.0 (35–41)
Weight at baseline, kg	
Mean (s.d.)	4.1 (0.53)
Median (range)	4.1 (3.10–5.20)
Sex, *n* (%)	
Male	6 (40)
Female	9 (60)
Race, *n* (%)	
White	10 (67)
Asian	2 (13)
Other	2 (13)
American Indian or Alaska Native	1 (7)
Ethnicity, *n* (%)	
Not Hispanic or Latino	13 (87)
Hispanic or Latino	2 (13)
Modality of SMA diagnosis, *n* (%)	
Prenatal testing	1 (7)
Newborn screening	13 (87)
Other	1 (7)
Age at SMA diagnosis, days^c^	
*n* (number of children diagnosed after birth)	14
Mean (s.d.)	9.9 (7.69)
Median (range)	8.0 (2–26)

^a^ITT population, *n* = 6 males and *n* = 9 females; mean (s.d.) age at dosing, 28.7 (11.68) days.

^b^Age at dosing = (dose date − date of birth + 1).

^c^Age at SMA diagnosis = (SMA diagnosis date − date of birth + 1). Only calculated for patients who were diagnosed after birth.

At screening, all included infants demonstrated normal neuromuscular function and were able to swallow and breathe normally. Median baseline peroneal CMAP was 4.10 mV (range, 2.7–7.0). All 15 infants enrolled in the three-copy cohort received the onasemnogene abeparvovec infusion at a median age of 32 days (range, 9–43), with a median baseline weight of 4.1 kg (range, 3.1–5.2). Infusion interruption occurred in one child because of a pump malfunction, but this child still received all of the intended dose. All children completed the study and were included in the intention-to-treat (ITT) population.

### Primary and secondary motor milestone endpoints

**Fig. 1:**
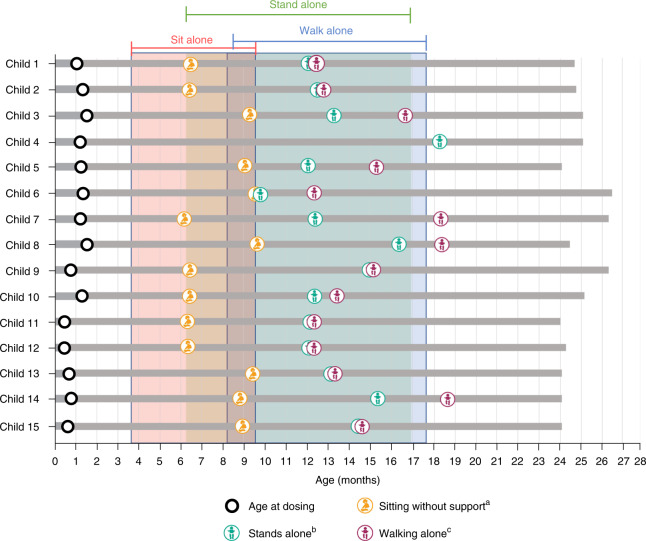
Video-confirmed developmental motor milestones for children with three copies of *SMN2*. Months calculated as days / 30. Only the first observed instance of a milestone is included in this figure. Shaded areas indicate the World Health Organization Multicentre Growth Reference Study (WHO-MGRS) windows for normal development; the 99th percentile (that is, upper bound of normal development) of sits without support is 279 days, stands alone is 514 days, and walks alone is 534 days. ^a^Bayley Scales gross motor subtest item #26: child sits alone without support for at least 30 seconds. ^b^Bayley Scales gross motor subtest item #40: child stands alone. Child stands alone for at least 3 seconds after you release his or her hands. ^c^Bayley Scales gross motor subtest item #43: child walks alone. Child takes at least five steps independently, displaying coordination and balance. *n* = 6 males and *n* = 9 females; mean (s.d.) age at dosing, 28.7 (11.68) days.

All 15 (100%) children achieved the primary endpoint of independent standing, confirmed by independent video review, for at least 3 seconds at any visit up to 24 months of age. Children achieved this motor milestone at a median age of 377 days (range, 284–549), and 14 of 15 (93%) did so within the normal WHO developmental window of ≤514 days (99th percentile) (Fig. 1). All children maintained this motor milestone at the 24-month study visit. For comparison, only 19 of 81 (24%) patients with SMA in the PNCR natural history population achieved independent standing (*P* < 0.0001)^14^.

Fourteen (93%) children in the three-copy cohort walked independently for at least five steps at any visit up to 24 months of age, compared to 17 of 81 patients (21%) in the PNCR population (*P* < 0.0001)^14^. The median age of independent walking was 422.0 days (range, 362–563), and 11 (73%) children achieved this motor milestone within the WHO normal developmental window of ≤534 days of age. Notably, one additional child in the three-copy cohort was observed walking during the 24-month study visit (conducted via video call) by the clinical evaluator, but video was not recorded. Therefore, per the study protocol, the child was judged not to have achieved this motor milestone in the absence of an independent video review. A detailed summary of motor milestones is included in Supplementary Table 2.

### Exploratory functional endpoints

**Fig. 2:**
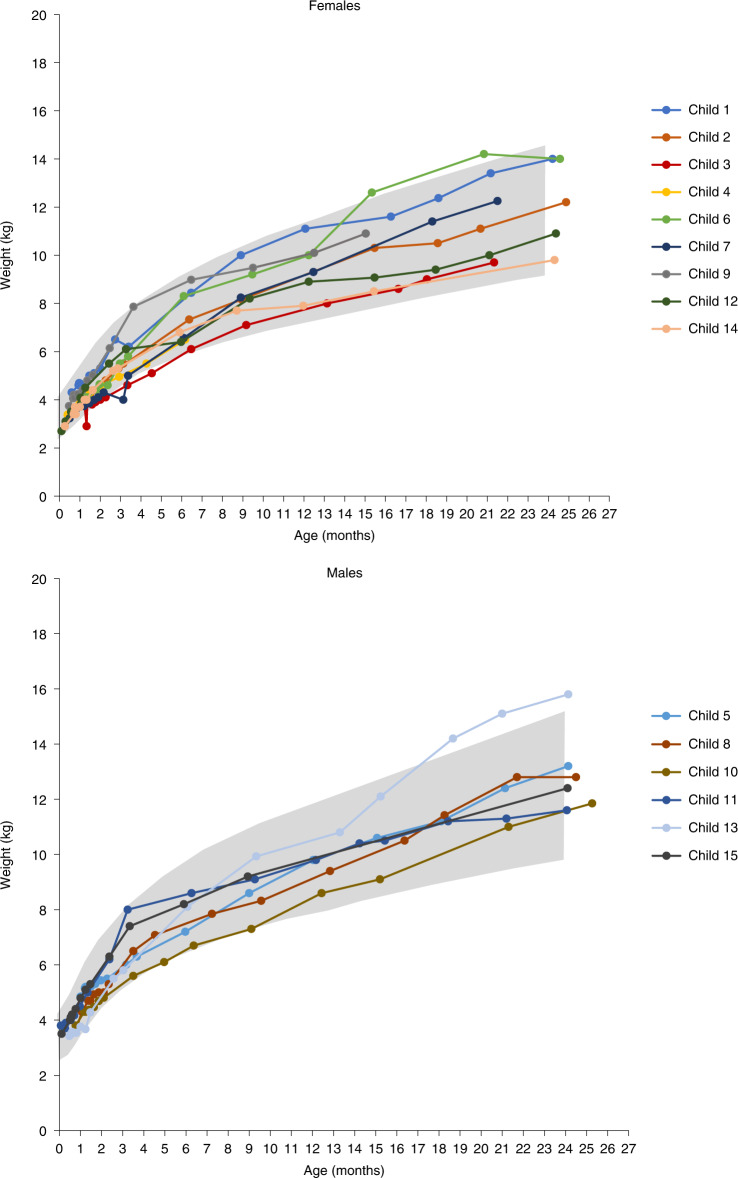
Growth charts for children with three copies of *SMN2*. Ten (67%) children achieved the ability to maintain weight at or above the WHO 3rd percentile without the need for non-oral/mechanical feeding support at all visits up to 24 months of age. The ability to maintain weight at or above the 3rd percentile without the need for non-oral/mechanical feeding support was defined by meeting both of the following criteria at all visits: (1) does not receive nutrition through mechanical support (that is, feeding tube) and (2) maintains weight (≥3rd percentile for age and sex as defined by WHO guidelines) consistent with the patient’s age at the assessment. The gray shading represents WHO growth standards for the 3rd through 97th percentiles. *n* = 6 males and *n* = 9 females; mean (s.d.) age at dosing, 28.7 (11.68) days.

All 15 (100%) children in the three-copy cohort were alive and free from permanent ventilation at 14 months of age, and ventilator-free survival remained 100% at the end of the study. In fact, no child required mechanical respiratory support of any kind (for example, cough-assist, bilevel positive airway pressure, or invasive ventilatory support) throughout the duration of the trial. Ten of 15 (67%) children were at or above the 3rd reference percentile for weight at all study visits, and all children were at or above this percentile at the end of the study (Fig. 2). In addition, no child required a feeding tube at any point during the study.

### Exploratory motor endpoints

**Fig. 3:**
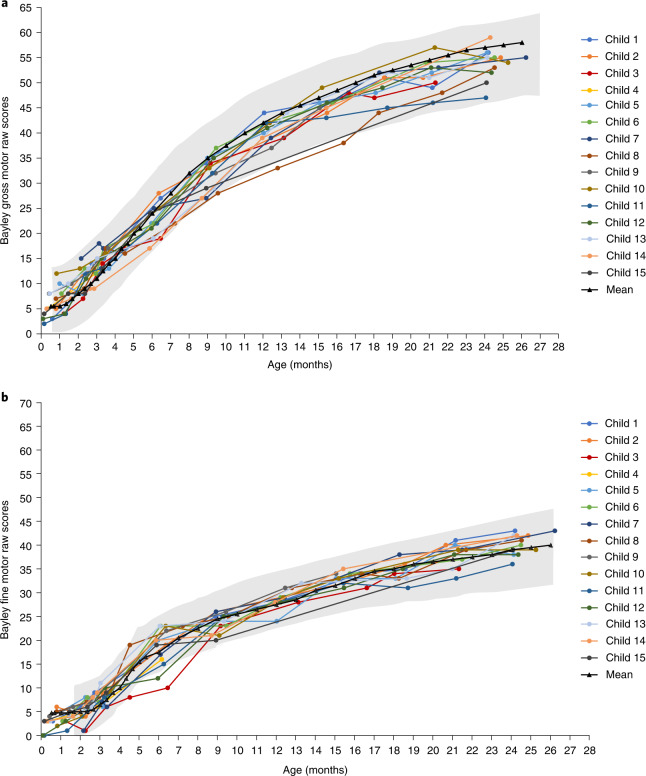
Bayley scales fine motor and gross motor raw scores. Improvements were observed in all children for both gross (**a**) and fine (**b**) subtests of the Bayley Scales of Infant and Toddler Development after onasemnogene abeparvovec infusion and up to 24 months of age. The gray shading represents Bayley-III gross and fine motor normal ranges (±2 s.d.). *n* = 6 males and *n* = 9 females; mean (s.d.) age at dosing, 28.7 (11.68) days.

The Bayley Scales of Infant and Toddler Development (BSID) provide a more granular appraisal of development compared with an age-matched reference population^33^. All 15 children were adequately assessed using the BSID, although one (7%) child missed the baseline assessment, precluding calculation of a change from baseline. Incremental gains in gross and fine motor raw scores generally tracked with the normal reference population (Fig. 3 and Supplementary Table 3). Raw scores were converted to scaled scores with a normative mean of 10 and a standard deviation (s.d.) of 3, such that scaled scores of 4–16 capture the 3rd to 97th percentile range for normal motor development. All 15 (100%) children in the three-copy cohort achieved a scaled score of ≥4 (within 2 s.d. of the reference mean) on both the gross motor and fine motor subtests during at least one post-baseline visit. For each scheduled visit, most assessed children (78–100%) met the criteria at that visit. At the 24-month visit, all ten (100%) children who were assessed achieved a scaled score of ≥4, with the median gross motor scaled score of 9 (range, 5–12), close to the normative population mean (Supplementary Table 4). Gains in motor function were paralleled by electrophysiologic evidence of preserved motor nerve integrity. The median maximum peroneal CMAP recorded at any post-infusion visit was 6.00 mV (range, 4.2–8.5), representing a median increase from baseline of 1.80 mV (range, −0.6 to 5.0) (Supplementary Table 5).

### Safety observations

To attenuate the inflammatory response to AAV9, all 15 children commenced oral prednisolone 1 day before onasemnogene abeparvovec infusion and completed a median of 63 days (range, 49–321) of therapy. A total of 166 treatment-emergent adverse events (TEAEs) were reported (Supplementary Tables 6 and 7). Each child experienced at least one TEAE, and three (20%) had a TEAE reported as serious. Eight of 15 (53%) children had a TEAE considered by the investigator to be related to the study treatment, but none was serious.

Transient decreases in platelets have been observed after administration of onasemnogene abeparvovec^34^. Preclinical studies in animal models have reported cardiac thrombi and dorsal root ganglia toxicities, but these have not been observed clinically^25^. On the basis of these data, the study sponsor (Novartis Gene Therapies) identified five specific categories of adverse events (AEs) of special interest (AESI): hepatotoxicity, thrombocytopenia, cardiac toxicity, thrombotic microangiopathy (TMA), and sensory abnormalities suggestive of dorsal root ganglionopathy (Table 2). AESIs were identified using specific terms in standardized Medical Dictionary for Regulatory Activities (MedDRA) coding dictionary queries related to these categories (see Methods for further details).

**Table 2:** TEAEs of special interest in children with three copies of *SMN2*

Category of AESI	*n* = 15^a^
Preferred term	*n* (%)
**Hepatotoxicity**	
Any TEAE	4 (27)
Aspartate aminotransferase increased	4 (27)
Alanine aminotransferase increased	3 (20)
Blood alkaline phosphatase increased	1 (7)
Gamma-glutamyltransferase increased	1 (7)
**Thrombocytopenia**	
Any TEAE	2 (13)
Hematemesis	1 (7)
Hematochezia	1 (7)
Contusion	1 (7)
**Cardiac adverse events**	
Any TEAE	3 (20)
Blood creatine phosphokinase MB increased	2 (13)
Troponin increased	2 (13)
**Thrombotic microangiopathy**	
Any TEAE	0 (0)
Thrombocytopenia	0 (0)
**Sensory abnormalities suggestive of dorsal root ganglionopathy**	
Any TEAE	1 (7)
Areflexia	1 (7)

^a^Safety population: *n* = 6 males and *n* = 9 females; mean (s.d.) age at dosing, 28.7 (11.68) days.

Thirteen hepatotoxicity AESIs occurred in four of 15 (27%) children. All events were mild or moderate, except for a single Grade 3 event of increased alanine aminotransferase (five or more times the upper limit of normal). The investigator considered all events as related to treatment. All hepatotoxicity events resolved, including the Grade 3 event that resolved with augmented prednisolone (Supplementary Table 8). No clinically observed events of jaundice or hepatic encephalopathy were reported. Three thrombocytopenia-related events occurred in two of 15 (13%) children. None of the events was associated with decreased platelet counts. These events were mild or moderate, considered unrelated to treatment, and resolved without sequelae (Supplementary Table 9). To assess cardiac toxicity, creatinine phosphokinase (CK)-MB was initially measured but was changed, mid-study, to the more reliable cardiac tissue marker troponin I. CK-MB was not assessed after this change. Eight children had both baseline and post-baseline CK-MB values, and five children had both baseline and post-baseline troponin I values. Four AEs of elevated cardiac enzymes were reported in three children: one had elevated CK-MB and troponin I; one had an isolated elevation of CK-MB; and one had an isolated elevation of troponin I (Table 2). All events were mild or moderate and considered possibly or probably related to treatment. At the end of the study, serum CK-MB remained elevated in one child, and the two cardiac AESIs resolved without sequelae in the other two children (Supplementary Table 10). No TMA events were reported during the study. One of 15 (7%) children had two AESIs (areflexia), which could potentially be related to dorsal root ganglionopathy. However, these events were mild and not related to treatment. One event resolved and one was ongoing at the last study visit (Supplementary Table 11).

## Discussion

SPR1NT demonstrates that a single intravenous dose of onasemnogene abeparvovec promotes motor development for presymptomatic neonates with biallelic deletions of *SMN1* and three copies of *SMN2* who are primarily at risk for SMA type 2. Without treatment, most of these children would achieve motor milestones no more advanced than independent sitting, whereas those treated with onasemnogene abeparvovec displayed patterns of motor development indistinguishable from healthy children without SMA. Specifically, all but one of the 15 children achieved independent walking (the remaining child stood independently), and all had BSID gross and fine motor scores similar to neurologically normal peers. Exceptional motor and functional outcomes were also observed for children in the two-copy cohort of SPR1NT^32^.

Remarkably, no child in SPR1NT required mechanical feeding or respiratory support, indicating that presymptomatic gene therapy has the potential to prevent some musculoskeletal, pulmonary, and growth complications characteristic of classic SMA type 2. This represents a profound shift in the early course of illness to a much milder SMA phenotype or possibly even to normal motor development. Given the durability of benefit observed in the follow-up study of the Phase I START trial^35^, and the fact that motor neurons are non-dividing cells, we are optimistic that one-time treatment with onasemnogene abeparvovec will add years of independent mobility, intact bulbar function, and good health-related quality of life for children in the three-copy cohort.

Table 3 places SPR1NT in the context of three other clinical trials: STR1VE-US^36^, STR1VE-EU^37^ and a Phase II study of infants with two or three copies of *SMN2* treated with nusinersen before symptom onset (NURTURE)^29^. A presymptomatic study with risdiplam (RAINBOWFISH) is still in progress^38^. Overall, Table 3 highlights the importance of treatment timing (that is, before the onset of clinical symptoms) as a potentially important determinant of outcome, but noteworthy differences in the designs of these trials prevent direct comparisons between them. Primary endpoints of the percentage of children who achieved the independent standing (BSID item #40) and independent walking (BSID item #43) motor milestones were included for the SPR1NT three-copy cohort versus only independent sitting (using WHO and Hammersmith Infant Neurological Examination section 2 criteria) in NURTURE. Eligibility criteria also differed, including ability to tolerate thin liquids, peroneal CMAP ≥2 mV, and presymptomatic SMA in SPR1NT versus ulnar CMAP ≥1 mV, absence of hypoxia, and no clinical signs or symptoms suggestive of SMA in NURTURE. Motor milestone achievement in both the two-copy and three-copy cohorts of SPR1NT is also distinguished from other studies by its stringency, requiring video confirmation by an independent observer in both the two-copy and three-copy cohorts. In NURTURE, however, parents or caregivers reported motor milestone achievement, and confirmation followed at the next site visit, and age at milestone achievement was reported by parents, caregivers, or site investigators. Regardless of these differences, children with few or no clinical signs of SMA who receive treatment appear to achieve more advanced developmental milestones than children who receive treatment after the clinical onset of disease.

**Table 3:** Summary of SPR1NT results and other SMA studies and cohorts^a^

		Onasemnogene abeparvovec				Nusinersen	
		Symptomatic patients		Presymptomatic children		Presymptomatic children	
	PNCR^14^	STR1VE-US^36^	STR1VE-EU^37^	SPR1NT, two-copy cohort	SPR1NT, three-copy cohort	NURTURE,^b^ two-copy cohort^30^	NURTURE,^b^ three-copy cohort^30^
Intention-to-treat population, *n*	23	22	32	14	15	15	10
*SMN2* copies	2	2	2	2	3	2	3
Median (range) age at diagnosis, days	N/A	67 (56–126)^c^	76 (26–156)	8 (1–14)	8 (2–26)	N/A	N/A
Median (range) age at infusion, days	N/A	105 (15–177)	123 (54–180)	21 (8–34)	32 (9–43)	19 (8–41)	23 (3–42)
Baseline median (range) CHOP INTEND	32.5 (31–33)^d^	33.5 (18–52)	28.0 (14–55)	48.5 (28–57)	N/A	45.0 (25–60)	53.5 (40–60)
Baseline median (range) CMAP amplitude, mV^e^	0.3 (0.04–1.1)	N/A	N/A	3.9 (2.1–6.1)	4.1 (2.7–7.0)	3.2 (1.1–9.7)	4.0 (0.2–7.0)
Sitting independently by 18 months, *n* (%)^f^	0	14 (64)	14 (44)	14 (100)	N/A	N/A	N/A
Sitting independently by 24 months of age, *n* (%)^f^	0	N/A	N/A	N/A	14 (93)	15 (100)	10 (100)
Standing independently by 18 months of age, *n* (%)^f^	0	1 (5)	1 (3)	11 (79)	N/A	N/A	N/A
Standing independently by 24 months of age, *n* (%)^f^	0	N/A	N/A	N/A	15 (100)	9 (60)	10 (100)
Walking independently by 18 months of age, *n* (%)^f^	0	1 (5)	1 (3)	9 (64)	N/A	N/A	N/A
Walking independently by 24 months of age, *n* (%)^f^	0	N/A	N/A	N/A	14 (93)	9 (60)	10 (100)
Alive without permanent ventilation at 18 months of age, *n* (%)^f^	6 (26)^g^	20 (91)	31 (97)	14 (100)	15 (100)	15 (100)	10 (100)

HINE-2, Hammersmith Infant Neurological Examination section 2; N/A, not available.

^a^There are no published head-to-head studies of onasemnogene abeparvovec and nusinersen. Differences in trial design, including primary endpoints, how endpoints were measured, and eligibility criteria, make direct comparison of results from these studies infeasible. The PNCR measured CHOP INTEND; NURTURE measured WHO and HINE-2 criteria; and STR1VE-US and STR1VE-EU measured WHO criteria and CHOP INTEND.

^b^NURTURE results represent interim analysis at data cut of 29 March 2019. At the time of this analysis, the median age of the infants was 34.8 months (range, 25.7–45.4)^26^.

^c^Median (range) is reported as the interquartile range.

^d^Value obtained for patients with symptom onset <3 months of age, including seven patients with two *SMN2* copies and one patient with three *SMN2* copies

^e^Ulnar CMAP amplitude recorded from the abductor digiti minimi muscle at baseline for the PNCR and NURTURE studies and peroneal CMAP amplitude recorded from the tibialis anterior muscle for SPR1NT.

^f^Milestones were evaluated over different observation periods between studies and included 18 months for STR1VE-US, STR1VE-EU, and SPR1NT two-copy cohort; 24 months for the SPR1NT three-copy cohort; and a median follow-up time of 35 months for NURTURE.

^g^Survival without permanent ventilation at 14 months.

We also observed that presymptomatic neonatal treatment with intravenous onasemnogene abeparvovec demonstrated a favorable safety profile, and no new or unexpected safety concerns were identified with treatment administration between 9 days and 43 days of age. Although all children had at least one AE, few experienced serious AEs, and no treatment-related serious AEs or deaths related to treatment were reported. Furthermore, AESIs were generally mild or moderate and resolved. Transient elevations of liver enzymes were asymptomatic and generally mild. Transient changes in platelet counts were observed, but no child had a platelet count below 75,000 cells per µl. All thrombocytopenia-related AESIs were mild to moderate, and all resolved. Asymptomatic and mild elevations of cardiac enzymes occurred in a minority of children but were not associated with signs of ventricular dysfunction or thrombosis. TMA, which presents clinically as hemolytic anemia, thrombocytopenia, and acute kidney injury, has been identified as a risk for onasemnogene abeparvovec based on post-marketing safety surveillance^39^, but no cases of TMA occurred in SPR1NT. A single case of areflexia persisted at the time of study conclusion, and, although areflexia is a component of the clinical picture of sensory ganglionopathy, other clinical symptoms of this condition were absent, increasing the likelihood that this AESI was a complication of one child’s underlying SMA diagnosis. The possibility that the favorable safety profile of onasemnogene abeparvovec observed in SPR1NT relates to maturational differences in the immune response is discussed briefly in the companion manuscript^32^. Limitations of SPR1NT are the relatively small number of participants, the use of an external comparator group, and the exclusion of participants with CMAP <2 mV at screening.

Two decades ago, the Human Genome Project promised new diagnostics and therapeutics based on the identification of underlying genetic mechanisms of disease^40^. Ultimately, genomics research aimed to change medical practice from a reactive stance, in which presenting signs and symptoms of disease prompt treatment, to a proactive one, in which deep understanding of underlying vulnerabilities within the genome allows providers to anticipate future health risks and apply precise interventions that keep people healthy. The goal was to find the right treatment for the right patient at the right time and, thereby, prevent disease and disability^41,42^. This goal may soon be realized for children with SMA, with the discovery of its molecular basis, effective therapies, and the optimal timing for intervention.

For all forms of SMA, genomic medicine appears to be entering the realms of public health and preventive pediatrics. Children at risk for SMA types 2 or 3 who were treated once with onasemnogene abeparvovec before 6 weeks of age, before the onset of symptoms, demonstrated normal or nearly normal patterns of growth and neuromuscular development in this study. Our findings underscore the urgency of early identification of children at risk for SMA by newborn screening, followed by timely treatment to prevent death and disability. This has critical implications for the implementation of universal newborn screening for *SMN1* deletions, discussed more fully in the companion two-copy manuscript^32^.

In the past 2 decades, advances in medical genetics have propelled the development of new therapies for monogenic disorders such as SMA, increased understanding of their underlying pathophysiology, and permitted development of new genetic diagnostic tools^42–44^. However, treating individuals who demonstrate no symptoms of disease remains controversial. SMA offers an example of what can be achieved when newborn screening identifies at-risk infants who can potentially be spared the consequences of severe, debilitating weakness. Children with three copies of *SMN2* have a greater likelihood of developing SMA type 2 or type 3, but SPR1NT demonstrates that treating three-copy children before the appearance of SMA symptoms essentially allows them to grow and develop as normal children. This represents a remarkable evolution in the standard of care for SMA: from a reactive to a proactive paradigm, from a focus on *patients* who survive to *children* who thrive.

## Methods

### Study design

SPR1NT was an open-label, single-arm, Phase III study conducted at 16 sites in six countries (Australia, Belgium, Canada, Japan, the United Kingdom and the United States). The study was conducted in accordance with the Declaration of Helsinki, International Council for Harmonisation/Good Clinical Practice guidelines and applicable regulatory requirements (for example, those relating to informed consent and the protection of human patients in biomedical research). The study was approved by institutional review boards (IRBs) at all participating institutions (Advarra Center for IRB Intelligence, Nationwide Children’s Hospital; UCLA Medical Center IRB #3, David Geffen School of Medicine at the University of California, Los Angeles; Nemours Office of Human Subjects Protection, Nemours Children’s Clinic; Columbia University Medical Center IRB, Columbia University Medical Center; Advarra Center for IRB Intelligence, Massachusetts General Hospital; Children’s Hospital of Eastern Ontario Research Ethics Board, Children’s Hospital of Eastern Ontario; Sydney Children’s Hospitals Network Human Research Ethics Committee, Sydney Children’s Hospital; University of Pennsylvania IRB, Clinic for Special Children; Tokyo Women’s Medical University IRB, Tokyo Women’s Medical University Hospital; The Dubowitz Neuromuscular Centre IRB, University College London; and The Neuromuscular Center of Liège, CHU & University of Liège), and written informed consent was obtained from parents or legal guardians of enrolled patients.

### Patients

The study included presymptomatic children who had SMA genetically defined by biallelic deletions of *SMN1* with either two or three copies of *SMN2* expected to develop SMA type 1 or SMA types 2 or 3, respectively. Children were enrolled in two separate cohorts according to the number of *SMN2* copies present. Children with *SMN1* point mutations (that is, pathogenic variants) or the *SMN2* gene modifier variant (c.859 G>C) could enroll, but those with the *SMN2* gene modifier variant would not be included in the ITT population. Efficacy and safety findings for children with three *SMN2* copies are reported. The study planned to enroll at least 12 children with three copies of *SMN2* who met the ITT criteria and were ≤6 weeks of age at the time of gene replacement therapy (Day 1). Full eligibility criteria are described in the Supplementary Material.

The Coronavirus Disease 2019 (COVID-19) pandemic did not affect retention. No participant withdrew from SPR1NT or was lost to follow-up because of the COVID-19 pandemic. However, some scheduled study visits and assessments were delayed or cancelled because of restrictions caused by the COVID-19 pandemic.

### Procedures

All children were admitted into the hospital for pretreatment baseline procedures 1 day before infusion. For all assessments, baseline was defined as the last assessment conducted before dosing. Onasemnogene abeparvovec (1.1 × 10^14^ vector genomes per kilogram (vg kg^−1^)) was administered as a single intravenous infusion (given over approximately 60 minutes) between 18 September 2018 and 9 July 2019. Safety monitoring was conducted while the children remained in the hospital for a minimum of 24 hours after infusion. All children received prophylactic prednisolone (initially 1 mg/kg/day, increased to 2 mg/kg/day following a protocol amendment in May 2019) beginning 24 hours before infusion and for 48 hours after infusion, after which the dosage was 1 mg/kg/day during a minimum of 30 days. Thereafter, prednisolone was tapered according to a standard algorithm and based on a requirement that gamma-glutamyltransferase, alanine aminotransferase, and aspartate aminotransferase values were below the threshold of twice the upper limit of normal. Investigators were permitted to use other glucocorticosteroids in place of prednisolone, change the daily prednisolone dosage, and alter the taper schedule according to their clinical judgment.

Outpatient follow-up assessments were conducted on Days 7, 14, 21, 30, 44, 51 (in Japan only), 60, and 72 post-dose, and then at 3 months of age and every 3 months thereafter through 24 months of age (that is, the end-of-study visit). All eligible children were invited to enroll in an ongoing long-term follow-up study (LT-002, NCT04042025).

### Outcomes

The primary efficacy endpoint was the ability to stand independently for ≥3 seconds at any visit up to 24 months of age, as stipulated by item #40 from the gross motor subtest of the BSID. The secondary efficacy endpoint was the ability to walk alone for at least five steps at any visit up to 24 months of age, as stipulated by item #43 of the BSID^33^. Exploratory endpoints were survival at 14 months of age, defined as the avoidance of death or requirement of permanent ventilation (tracheostomy or ≥16 hours of daily respiratory assistance for ≥14 consecutive days in the absence of an acute reversible illness, excluding perioperative ventilation) and the ability to maintain body weight at or above the 3rd percentile without the need for feeding support at any visit up to 24 months of age. Other exploratory endpoints included achievement of motor milestones and changes from baseline as assessed by World Health Organization Multicentre Growth Reference Study (WHO-MGRS) and BSID version 3 gross motor criteria, Children’s Hospital of Pennsylvania Infant Test of Neuromuscular Disorders (CHOP INTEND) scores, and scores on the BSID gross and fine motor subtests. Videos demonstrating developmental milestones meeting WHO and BSID criteria (as part of clinical evaluation at study visits or submitted by parent(s)/legal guardian(s) at any time during the study) were reviewed by an independent, central reviewer for unbiased assessment and confirmation of developmental milestone achievement.

### Safety monitoring

Safety was assessed by monitoring for AEs, physical examinations, pulmonary examinations, vital signs, weight and length measurements, 12-lead electrocardiograms, 24-hour Holter monitoring, echocardiograms, swallowing tests, laboratory assessments, and photographs of the infusion site. Pulmonary examinations were performed by a pulmonologist or appropriate individual according to standard institutional practice. All AEs were recorded and classified in accordance with the Common Terminology Criteria for Adverse Events (version 4.03) (https://www.eortc.be/services/doc/ctc/ctcae_4.03_2010-06-14_quickreference_5×7.pdf).

### Statistical analysis

Data were analyzed using SAS version 9.4 software (SAS Institute). Primary and secondary efficacy analyses were performed for participants with biallelic *SMN1* deletions and three copies of *SMN2* without the *SMN2* gene modifier variant (c.859 G>C), which is associated with a less severe clinical course^45^, who were included in the ITT population. Primary and secondary outcomes were compared with a cohort of population-matched patients from the PNCR natural history data set (all patients with any type of SMA and three copies of *SMN2*; the *SMN2* modifier mutation (c.859 G>C) was not assessed in the PNCR study cohort)^14^. This study was designed to have >90% power with α = 0.05 to detect a significant difference in independent standing using a two-sided Fisher exact test on a sample size of ≥12 children into the ITT population as well as assumptions based on a matched PNCR dataset^14^ and START study data^17^. Formal testing for the primary and secondary efficacy endpoints was performed using a hierarchical approach to protect against Type I error as follows. First, the primary motor endpoint of independent standing for ≥3 seconds was assessed. If the analysis of the primary endpoint was determined to be statistically significant (*P* < 0.05), then formal testing of the secondary motor endpoint, walking independently, was conducted.

The safety population included all children who received onasemnogene abeparvovec. Safety was evaluated through reported AEs as well as objective data variables, including vital signs, physical examinations, and laboratory studies. These data are presented in a descriptive fashion. AEs were coded using an industry standardized MedDRA coding dictionary (version 23.0), and AESIs were classified through specific predefined MedDRA terms (Supplementary Table 12).

### Reporting summary

Further information on research design is available in the Nature Research Reporting Summary linked to this article.

## Online content

Any methods, additional references, Nature Research reporting summaries, source data, extended data, supplementary information, acknowledgements, peer review information; details of author contributions and competing interests; and statements of data and code availability are available at 10.1038/s41591-022-01867-3.

## Supplementary information

Supplementary Information SPR1NT Study Group, Supplementary Methods, Supplementary Fig. 1 and Supplementary Tables 1–12

Reporting Summary

## Data Availability

A redacted version of the SPR1NT study protocol and a redacted version of the statistical analysis plan are available at ClinicalTrials.gov (NCT03505099). Novartis is committed to sharing clinical trial data with external researchers and has been doing so voluntarily since 2014. Novartis is committed to sharing, upon requests from qualified external researchers and subsequent approval by an independent review panel based upon scientific merit, anonymized patient-level and study-level clinical trial data and redacted clinical study reports for medicines and indications approved in the United States and Europe after the respective study is accepted for publication. All data provided are anonymized to respect the privacy of patients who have participated in the trial, in line with applicable laws and regulations. This trial data availability is according to the criteria and process described on www.clinicalstudydatarequest.com.

## References

[1] Coovert DD (1997). The survival motor neuron protein in spinal muscular atrophy. Hum. Mol. Genet..

[2] Mailman MD (2002). Molecular analysis of spinal muscular atrophy and modification of the phenotype by SMN2. Genet. Med..

[3] Calucho M (2018). Correlation between SMA type and *SMN2* copy number revisited: an analysis of 625 unrelated Spanish patients and a compilation of 2834 reported cases. Neuromuscul. Disord..

[4] Carson VJ (2022). Nusinersen by subcutaneous intrathecal catheter for symptomatic spinal muscular atrophy patients with complex spine anatomy. Muscle Nerve.

[5] Muntoni F (2020). Long-term follow-up of patients with type 2 and non-ambulant type 3 spinal muscular atrophy (SMA) treated with olesoxime in the OLEOS trial. Neuromuscul. Disord..

[6] D’Amico A, Mercuri E, Tiziano FD, Bertini E (2011). Spinal muscular atrophy. Orphanet J. Rare Dis..

[7] Chabanon A (2018). Prospective and longitudinal natural history study of patients with type 2 and 3 spinal muscular atrophy: baseline data NatHis-SMA study. PLoS ONE.

[8] Kaufmann P (2012). Prospective cohort study of spinal muscular atrophy types 2 and 3. Neurology.

[9] Trucco F (2021). Respiratory trajectories in type 2 and 3 spinal muscular atrophy in the iSMAC cohort study. Neurology.

[10] Coratti G (2021). Motor function in type 2 and 3 SMA patients treated with nusinersen: a critical review and meta-analysis. Orphanet J. Rare Dis..

[11] Mercuri E (2016). Patterns of disease progression in type 2 and 3 SMA: implications for clinical trials. Neuromuscul. Disord..

[12] Annoussamy M (2021). Natural history of type 2 and 3 spinal muscular atrophy: 2-year NatHis-SMA study. Ann. Clin. Transl. Neurol..

[13] Farrar MA (2013). Pathophysiological insights derived by natural history and motor function of spinal muscular atrophy. J. Pediatr..

[14] Finkel RS (2014). Observational study of spinal muscular atrophy type I and implications for clinical trials. Neurology.

[15] Swoboda KJ (2005). Natural history of denervation in SMA: relation to age, *SMN2* copy number, and function. Ann. Neurol..

[16] Ramdas S, Servais L (2020). New treatments in spinal muscular atrophy: an overview of currently available data. Expert Opin. Pharmacother..

[17] Mendell JR (2017). Single-dose gene-replacement therapy for spinal muscular atrophy. N. Engl. J. Med..

[18] Hale K (2021). Landscape of spinal muscular atrophy newborn screening in the United States: 2018–2021. Int. J. Neonatal Screen..

[19] Kariyawasam DST (2020). The implementation of newborn screening for spinal muscular atrophy: the Australian experience. Genet. Med..

[20] Jedrzejowska M (2020). Advances in newborn screening and presymptomatic diagnosis of spinal muscular atrophy. Degener. Neurol. Neuromuscul. Dis..

[21] Friese J (2021). Safety monitoring of gene therapy for spinal muscular atrophy with onasemnogene abeparvovec—a single centre experience. J. Neuromuscul. Dis..

[22] Gaber Ali H (2021). Gene therapy for spinal muscular atrophy: the Qatari experience. Gene Ther..

[23] Waldrop MA (2020). Gene therapy for spinal muscular atrophy: safety and early outcomes. Pediatrics.

[24] Weiβ C (2022). Gene replacement therapy with onasemnogene abeparvovec in children with spinal muscular atrophy aged 24 months or younger and bodyweight up to 15 kg: an observational cohort study. Lancet Child Adolesc. Health.

[25] Day JW (2021). Clinical trial and postmarketing safety of onasemnogene abeparvovec therapy. Drug Saf..

[26] D’Silva AM (2022). Onasemnogene abeparvovec in spinal muscular atrophy: an Australian experience of safety and efficacy. Ann. Clin. Transl. Neurol..

[27] Servais, L. et al. Real-world treatment patterns and outcomes in patients with spinal muscular atrophy: updated findings from the RESTORE registry. Presented at: World Muscle Society 2021 Congress, 20–24 September 2021; Virtual.

[28] Servais, L. et al. The RESTORE Registry: real-world assessments of interventions and long-term outcomes in patients with spinal muscular atrophy. Presented at: British Paediatric Neurology Association 2022 Annual Conference, 19–21 January 2022; Virtual.

[29] Servais, L. et al. Effectiveness and safety of onasemnogene abeparvovec in older patients with spinal muscular atrophy (SMA): real-world outcomes from the RESTORE Registry. Presented at: British Paediatric Neurology Association 2022 Annual Conference, 19–21 January 2022; Virtual.

[30] De Vivo DC (2019). Nusinersen initiated in infants during the presymptomatic stage of spinal muscular atrophy: interim efficacy and safety results from the phase 2 NURTURE study. Neuromuscul. Disord..

[31] WHO Multicentre Growth Reference Study Group. (2006). WHO Motor Development Study: windows of achievement for six gross motor development milestones. Acta Paediatr. Suppl..

[32] Strauss, K. A. et al. Onasemnogene abeparvovec for presymptomatic infants with two copies of SMN2 at risk for spinal muscular atrophy: The Phase III SPR1NT trial. *Nat. Med.*10.1038/s41591-022-01866-4 (2022). 10.1038/s41591-022-01867-3 PMC9205287 35715567

[33] Bayley, N. *Bayley Scales of Infant and Toddler Development: Administration Manual* 3rd edn (Pearson PsychCorp, 2006).

[34] Mendell JR (2021). Current clinical applications of in vivo gene therapy with AAVs. Mol. Ther..

[35] Mendell JR (2021). Five-year extension results of the phase 1 START trial of onasemnogene abeparvovec in spinal muscular atrophy. JAMA Neurol..

[36] Day JW (2021). Onasemnogene abeparvovec gene therapy for symptomatic infantile-onset spinal muscular atrophy in patients with two copies of *SMN2* (STR1VE): an open-label, single-arm, multicentre, phase 3 trial. Lancet Neurol..

[37] Mercuri E (2021). Onasemnogene abeparvovec gene therapy for symptomatic infantile-onset spinal muscular atrophy type 1 (STR1VE-EU): an open-label, single-arm, multicentre, phase 3 trial. Lancet Neurol..

[38] Finkel RS (2021). RAINBOWFISH: a study of risdiplam in newborns with presymptomatic spinal muscular atrophy (SMA). Neurology.

[39] Chand D (2021). Thrombotic microangiopathy following onasemnogene abeparvovec for spinal muscular atrophy: a case series. J. Pediatr..

[40] International Human Genome Sequencing Consortium. (2001). Initial sequencing and analysis of the human genome. Nature.

[41] Guttmacher AE, Jenkins J, Uhlmann WR (2001). Genomic medicine: who will practice it? A call to open arms. Am. J. Med. Genet..

[42] Hall WD, Mathews R, Morley KI (2010). Being more realistic about the public health impact of genomic medicine. PLoS Med..

[43] Lander ES (2011). Initial impact of the sequencing of the human genome. Nature.

[44] Green ED, Guyer MS (2011). National Human Genome Research Institute. Charting a course for genomic medicine from base pairs to bedside. Nature.

[45] Prior TW (2009). A positive modifier of spinal muscular atrophy in the *SMN2* gene. Am. J. Hum. Genet..

