# Supplementary material for: Onasemnogene abeparvovec for presymptomatic infants with three copies of SMN2 at risk for spinal muscular atrophy: the Phase III SPR1NT trial
Source: Nat Med. 2022 Jun 17;28(7):1390–7. doi: 10.1038/s41591-022-01867-3 (PMC9205287; doi:10.1038/s41591-022-01867-3)
Supplement: Supplementary file 1 — SPR1NT Study Group, Supplementary Methods, Supplementary Fig. 1 and Supplementary Tables 1–12 [file 41591_2022_1867_MOESM1_ESM.pdf]

nature portfolio

https://doi.org/10.1038/s41591-022-01867-3

Supplementary information

Onasemnogene abeparvovec for presymptomatic infants with three copies of *SMN2* at risk for spinal muscular atrophy: the Phase III SPR1NT trial

In the format provided by the authors and unedited

Table of Contents

SUPPLEMENTAL MATERIAL **2**
SPR1NT STUDY GROUP **2**
SUPPLEMENTARY METHODS **3**
Inclusion Criteria 3
Exclusion Criteria 3
SUPPLEMENTAL FIGURES **5**
Supplemental Figure 1. Patient disposition. 5
SUPPLEMENTARY TABLES **6**
Supplemental Table 1. Screen failures for the SPR1NT study 6
Supplemental Table 2. Age at which video-confirmed developmental milestones were achieved by children during study 7
Supplemental Table 3. Bayley raw and scaled scores at all time points 10
Supplemental Table 4. Percentage of children achieving a scaled score on Bayley gross and fine motor subtests ≥4 (±2 SD) up to 24 months of age 12
Supplemental Table 5. Summary of changes from baseline to maximum post-baseline values in CMAP (ITT population) 13
Supplemental Table 6. Treatment-emergent adverse events observed for two or more children 14
Supplemental Table 7. Treatment-emergent adverse events by system organ class 15
Supplemental Table 8. Hepatotoxicity-related treatment-emergent adverse events of special interest 16
Supplemental Table 9. Thrombocytopenia-related treatment-emergent adverse events of special interest 18
Supplemental Table 10. Cardiac treatment-emergent adverse events of special interest 19
Supplemental Table 11. Sensory abnormalities suggestive of ganglionitis adverse events of special interest 20
Supplemental Table 12. Summary of identification of adverse events of special interest 21

SUPPLEMENTAL MATERIAL

SPR1NT STUDY GROUP

The Dubowitz Neuromuscular Centre, University College London, Great Ormond Street Institute of Child Health & Great Ormond Street Hospital, London, UK
Mariacristina Scoto

Tokyo Women's Medical University, Tokyo, Japan
Tetsuo Ikai, Tamaki Kato, Satoru Nagata, Masaki Wada, Mari Matsuo, Masaki Ogawa, Eriko Shimada, Keiko Ishigaki, Hayato Suzuki (physiotherapist), Naoko Shima (physiotherapist), Kaho Nakamura (physiotherapist)

Sydney Children's Hospital Network, Randwick, NSW, Australia
Hugo Sampaio (sub-investigator), Professor Ian E. Alexander (sub-investigator), Jonathan Forsey (cardiologist), Karen Herbert (physiotherapist), Peter Barclay (pharmacist), Nicole Kerly (study coordinator), Stephanie Richardson (study coordinator)

The Neuromuscular Center of Liège, CHU & University of Liège, Liège, Belgium
Aurore Daron and Laura Vandenbrande (co-investigators), Fabian Dal Farra (physiotherapist), Olivier Schneider (physiotherapist), Alexa Jonas (pharmacist), Laura Buscemi (study coordinator)

SUPPLEMENTARY METHODS

Inclusion Criteria

Infants eligible for enrollment in the three-copy cohort of SPR1NT must have been genetically diagnosed with presymptomatic spinal muscular atrophy (SMA) with three copies of *SMN2*, ≤6 weeks (≤42 days) of age at the time of treatment, were able to tolerate thin liquids as demonstrated through a formal bedside swallowing test, had a baseline peroneal nerve to tibialis anterior compound muscle action potential (CMAP) value of ≥2 mV, were at a gestational age of 35 to 42 weeks, were up-to-date on childhood vaccinations that include palivizumab prophylaxis (also known as Synagis®) to prevent respiratory syncytial virus infections, able and willing to follow the Consensus Statement for Standard of Care in Spinal Muscular Atrophy and parent(s)/legal guardian(s) willing and able to complete the informed consent process and comply with study procedures and visit schedule. Genetic diagnoses had to be obtained from an acceptable newborn or prenatal screening test method.

Exclusion Criteria

Infants were excluded from enrollment for any of the following: (1) weight at screening visit <2 kg; (2) hypoxemia (oxygen saturation <96% awake or asleep without any supplemental oxygen or respiratory support) at the screening visit or for altitudes >1000 m, oxygen saturation of <92% awake or asleep without any supplemental oxygen or respiratory support at the screening visit; (3) any clinical signs or symptoms at screening or immediately prior to dosing that were, in the opinion of the investigator, strongly suggestive of SMA (e.g., tongue fasciculation, hypotonia, areflexia); (4) tracheostomy or current prophylactic use or requirement of non-invasive ventilatory support at any time and for any duration prior to screening or during the screening period; (5) children who had signs of aspiration/inability to tolerate non-thickened liquids based on a formal swallowing test performed as part of screening or patients receiving any non-oral feeding method; (6) children who had clinically significant abnormal laboratory values (gamma-glutamyltransferase, alanine aminotransferase, and aspartate aminotransferase, or total bilirubin >2× the upper limit of normal, creatinine ≥1.0 mg/dL, hemoglobin [Hgb] <8 or >18 g/dL; white blood cell [WBC] >20,000 per cmm) prior to gene replacement therapy; children with an elevated bilirubin concentration that was unequivocally the result of neonatal jaundice were not excluded; (7) children who demonstrated any other clinically significant abnormalities in hematology or clinical chemistry parameters as determined by the investigator or medical monitor; (8) children treated with an investigational or commercial product, including nusinersen, administered for the treatment of SMA; this included any history of gene therapy, prior antisense oligonucleotide treatment, or cell transplantation; (9) children whose weight-for-age was below the 3rd percentile based on World Health Organization (WHO) Child Growth Standards; (10) biologic mother with active viral infection as determined by screening laboratory samples (includes human immunodeficiency virus [HIV] or positive serology for hepatitis B or C); biologic mothers with a clinical suspicion of Zika virus that met Centers for Disease Control and Prevention (CDC) Zika virus epidemiological criteria, including a history of residence in or travel to a geographic region with active Zika transmission at the time of travel were tested for Zika virus RNA; positive results warrant confirmed negative Zika virus RNA testing for the child prior to enrollment; (11) serious non-respiratory tract illness requiring systemic treatment and/or hospitalization within 2 weeks prior to screening; (12) upper or lower respiratory infection that required medical attention, medical intervention, or an increase in supportive care

of any manner within 4 weeks prior to dosing; (13) severe nonpulmonary/respiratory tract infection (e.g., pyelonephritis or meningitis) within 4 weeks prior to administration of gene replacement therapy or concomitant illness that, in the opinion of the investigator or sponsor medical monitor, created unnecessary risks for gene replacement therapy, such as major renal or hepatic impairment, known seizure disorder, diabetes mellitus, idiopathic hypocalciuria, and symptomatic cardiomyopathy; (14) known allergy or hypersensitivity to prednisolone or other glucocorticosteroids or their excipients; (15) a previous, planned, or expected major surgical procedure, including scoliosis repair surgery/procedure during the study assessment period; (16) concomitant use of any of the following: drugs for treatment of myopathy or neuropathy, agents used to treat diabetes mellitus, or ongoing immunosuppressive therapy, plasmapheresis, immunomodulators such as adalimumab, immunosuppressive therapy within 4 weeks prior to gene replacement therapy (e.g., corticosteroids, cyclosporine, tacrolimus, methotrexate, cyclophosphamide, intravenous immunoglobulin, rituximab); (17) anti-AAV9 antibody titer >1:50 as determined by enzyme-linked immunosorbent assay (ELISA) binding immunoassay; if a potential child demonstrated anti-AAV9 antibody titer >1:50, he or she received retesting inside the 30-day screening period and were eligible to participate if the anti-AAV9 antibody titer upon retesting was ≤1:50, provided child was still <6 weeks of age at the time of dosing; (18) biologic mother was involved with the care of the child refused anti-AAV9 antibody testing prior to dosing; (19) parent(s)/legal guardian(s) who were unable or unwilling to comply with study procedures or the inability to travel for repeat visits; (20) parent(s)/legal guardian(s) who were unwilling to keep study results/observations confidential or to refrain from posting confidential study results/observations on social media sites; and (21) parent(s)/legal guardian(s) refused to sign consent form.

SUPPLEMENTAL FIGURES

Supplemental Figure 1. Patient disposition.

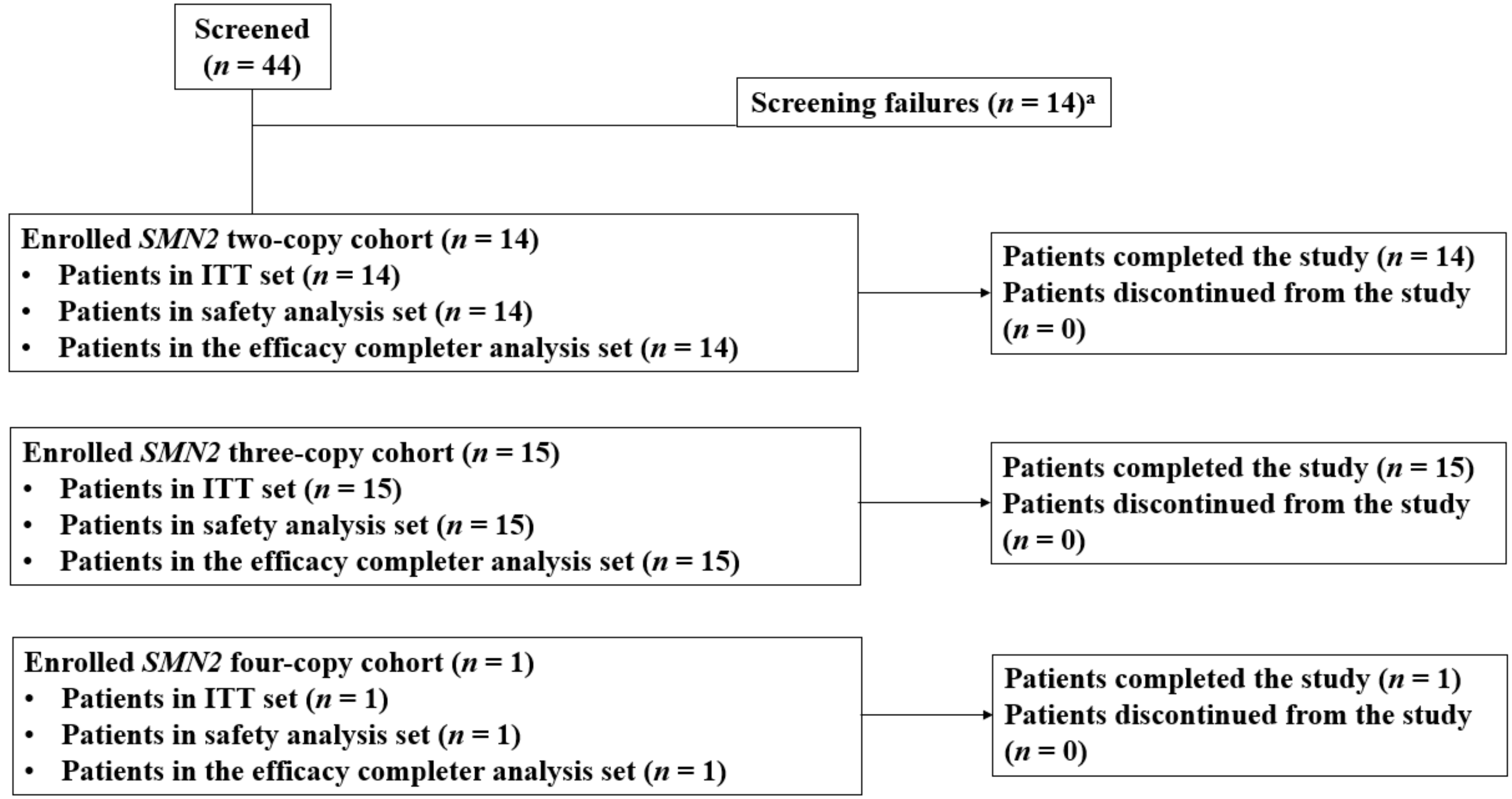

ITT, intention-to-treat; *SMN2*, *survival motor neuron 2* gene.
[a]Screen failures for both the two- and three-copy cohorts.

SUPPLEMENTARY TABLES

Supplemental Table 1. Screen failures for the SPR1NT study

**Criteria**	**Excluded patients (*n* = 14 total)[a]**
Clinical signs of SMA at screening	*n* = 4[b]
Baseline CMAP[c] <2 mV	*n* = 4
Elevated anti-AAV9 titers	*n* = 2
Clinically significant abnormal laboratory parameters	*n* = 1
Signs of aspiration/inability to tolerate non-thickened liquids based on a formal swallowing test	*n* = 1
Weight for age below 3rd percentile for age	*n* = 1
Lack of a genetic diagnosis, obtained from an acceptable newborn or pre-natal screening test method	*n* = 1
Parent(s)/legal guardian(s) unwilling or unable to complete the informed consent process and comply with study procedures and visit schedule	*n* = 1
Serious nonrespiratory tract illness requiring systemic treatment and/or hospitalization within 2 weeks prior to screening	*n* = 1

AAV9, adeno-associated virus-9; CMAP, compound muscle action potential; HIV, human immunodeficiency virus; SMA, spinal muscular atrophy.

[a]Screen failures for both SPR1NT cohorts.

[b]Two patients had two exclusionary criteria each (clinical signs of SMA at screening and abnormal swallow for one patient and clinical signs of SMA at screening and tibialis anterior CMAP <2 mV for the other patient).

[c]CMAP measured from peroneal nerve to tibialis anterior.

Supplemental Table 2. Age at which video-confirmed developmental milestones were achieved by children during study

	Age at dosing/Age at study completion (days)	Head control[a] Age (days/ months)	Rolls from back to sides[b] Age (days/ months)	Sits without support Age (days/months)		Crawls Age (days/months)		Stands with assistance Age (days/months)		Pulls to stand[i] Age (days/ months)	Stands alone[j,k] Age (days/ months)	Walks with assistance[l,m] Age (days/ months)	Walks alone[n,o] Age (days/ months)
				At least 30 secs[c]	At least 10 secs[d]	At least 5 feet[e]	Hands and knees crawling[f]	Supports weight 2 secs[g]	Holding stable object[h]				
Patient 1	29/742	√<br>60/2.0	√<br>194/6.5	√<br>194/6.5	√<br>194/6.5	√<br>362/12.1	√<br>362/12.1	√<br>267/8.9	√<br>267/8.9	√<br>267/8.9	√<br>362/12.1[j]<br>362/12.1[k]	√<br>362/12.1[l]<br>362/12.1[m]	√<br>362/12.1<br>362/12.1[o]
Patient 2	39/746	√<br>68/2.3	√<br>191/6.4	√<br>191/6.4	√<br>191/6.4	√<br>268/8.9	√<br>268/8.9	√<br>191/6.4	√<br>268/8.9	√<br>268/8.9	√<br>377/12.6[j]<br>377/12.6[k]	√<br>268/8.9[l]<br>268/8.9[m]	√<br>377/12.6<br>377/12.6
Patient 3	41/755	√<br>100/3.3	√<br>275/9.2	√<br>275/9.2	√<br>275/9.2	√<br>275/9.2	√<br>275/9.2	√<br>275/9.2	√<br>275/9.2	√<br>275/9.2	√<br>394/13.1[j]<br>499/16.6[k]	√<br>394/13.1[l]<br>394/13.1[m]	√<br>499/16.6[n]<br>499/16.6[o]
Patient 4	32/753	√<br>128/4.3	√<br>185/6.2								√<br>549/18.3[j]<br>549/18.3[k]		
Patient 5	37/724	√<br>At screening[p]	√<br>179/6.0	√<br>270/9.0	√<br>270/9.0	√<br>270/9.0	√<br>270/9.0	√<br>270/9.0	√<br>270/9.0	√<br>270/9.0	√<br>361/12.0[j]<br>361/12.0[k]	√<br>361/12.0[l]<br>361/12.0[m]	√<br>452/15.1[n]<br>452/15.1[o]
Patient 6	40/794	√<br>At screening[p]	√<br>284/9.5	√<br>284/9.5	√<br>284/9.5	√<br>284/9.5	√<br>284/9.5	√<br>284/9.5	√<br>284/9.5	√<br>284/9.5	√<br>284/9.5[j]<br>367/12.2[k]	√<br>284/9.5[l]<br>284/9.5[m]	√<br>367/12.2[n]<br>367/12.2[o]
Patient 7	37/787	√<br>65/2.2	√<br>183/6.1	√<br>183/6.1	√<br>183/6.1	373/12.4	√<br>373/12.4	√<br>373/12.4	√<br>268/8.9	√<br>373/12.4	√<br>373/12.4[j]<br>373/12.4[k]	√<br>373/12.4[l]<br>373/12.4[m]	√<br>549/18.3[n]<br>549/18.3[o]
Patient 8	43/737	√<br>At screening[p]	√<br>217/7.2	√<br>287/9.6	√<br>287/9.6	√<br>384/12.8	√<br>491/16.4	√<br>384/12.8	√<br>384/12.8	√<br>491/16.4	√<br>491/16.4[j]<br>548/18.3[k]	√<br>491/16.4[l]<br>491/16.4[m]	√<br>548/18.3[n]<br>491/16.4[o]
Patient 9[q]	21/787	√<br>At screening[p]	√<br>283/9.5	√<br>192/6.4	√<br>192/6.4	√<br>374/12.5	√<br>374/12.5	√<br>283/9.5	√<br>283/9.5	√<br>374/12.5	√<br>452/15.1[j]<br>452/15.1[k]	√<br>374/12.5[l]<br>374/12.5[m]	√<br>452/15.1[n]<br>452/15.1[o]
Patient 10	36/758	√<br>At screening[p]	√<br>456/15.2	√<br>191/6.4	√<br>191/6.4	√<br>273/9.1	√<br>273/9.1	√<br>273/9.1	√<br>273/9.1	√<br>273/9.1	√<br>371/12.4[j]<br>371/12.4[k]	√<br>371/12.4[l]<br>371/12.4[m]	√<br>401/13.4[n]<br>371/12.4[o]

	Age at dosing/Age at study completion (days)	Head control[a] Age (days/ months)	Rolls from back to sides[b] Age (days/ months)	Sits without support Age (days/months)		Crawls Age (days/months)		Stands with assistance Age (days/months)		Pulls to stand[i] Age (days/ months)	Stands alone[j,k] Age (days/ months)	Walks with assistance[l,m] Age (days/ months)	Walks alone[n,o] Age (days/ months)
				At least 30 secs[c]	At least 10 secs[d]	At least 5 feet[e]	Hands and knees crawling[f]	Supports weight 2 secs[g]	Holding stable object[h]				
Patient 11	9/722	√<br>71/2.4	√<br>636/21.2	√<br>188/6.3	√<br>188/6.3	√<br>278/9.3	√<br>278/9.3	√<br>278/9.3	√<br>278/9.3	√<br>365/12.2	√<br>365/12.2<br>365/12.2	√<br>365/12.2[l]<br>463/15.4[m]	√<br>365/12.2[n]<br>365/12.2[o]
Patient 12	10/731	√<br>38/1.3	√<br>234/7.8	√<br>190/6.3	√<br>280/9.3	√<br>268/8.9	268/8.9	√<br>268/8.9	280/9.3	√<br>268/8.9	√<br>367/12.2[j]<br>464/15.5[k]	√<br>367/12.2[l]<br>280/9.3[m]	√<br>367/12.2[n]<br>367/12.2[o]
Patient 13	18/724	√<br>At screening[p]	√<br>280/9.3	√<br>280/9.3	√<br>280/9.3	√<br>399/13.3	√<br>399/13.3	√<br>280/9.3	√<br>280/9.3	√<br>399/13.3	√<br>399/13.3[j]<br>399/13.3[k]	√<br>280/9.3[l]<br>399/13.3[m]	√<br>399/13.3[n]<br>399/13.3[o]
Patient 14	22/729	√<br>49/1.6	√<br>261/8.7	√<br>261/8.7	√<br>261/8.7	√<br>359/12.0	√<br>359/12.0	√<br>359/12.0	√<br>359/12.0	√<br>359/12.0	√<br>463/15.4[j]<br>463/15.4[k]	√<br>359/12.0[l]<br>359/12.0[m]	√<br>563/18.8[n]<br>463/15.4[o]
Patient 15	16/723	√<br>44/1.5	√<br>177/5.9	√<br>268/8.9	√<br>268/8.9	√<br>366/12.2	√<br>366/12.2	√<br>268/8.9	√<br>366/12.2	√<br>366/12.2	√<br>443/14.8[j]<br>443/14.8[k]	√<br>366/12.2[l]<br>366/12.2[m]	√<br>443/14.8[n]<br>443/14.8[o]

√ = Milestone achieved (Visit month identified); developmental milestones were video confirmed by an independent central reviewer.

Note: Months calculated as days/30. Only the first observed instance of a milestone is included in this table. Per WHO-MGRS definition, months calculated as days/30.4375.

Note: n = 6 males and n = 9 females; mean (SD) age at dosing, 28.7 (11.68) days.

[a]Bayley gross motor subtest item #4: Child holds head erect for at least 3 seconds without support.

[b]Bayley gross motor subtest item #20: Child turns from back to both right and left sides.

[c]Bayley gross motor subtest item #26: Child sits alone without support for at least 30 seconds.

[d]WHO-MGRS definition: Sitting without support. Child sits up straight with head erect for at least 10 seconds. Child does not use arms or hands to balance body or support position.

[e]Bayley gross motor subtest item #34: Child makes forward progress of at least 5 feet by crawling on hands and knees.

[f]WHO-MGRS definition: Hands-and-knees crawling. Child alternately moves forward or backward on hands and knees. The stomach does not touch the supporting surface. There are continuous and consecutive movements, at least 3 in a row.

[g]Bayley gross motor subtest item #33: Supports weight. Child supports his or her own weight for at least 2 seconds, using your hands for balance only.

[h]WHO-MGRS definition: Standing with assistance. Child stands in upright position on both feet, holding onto a stable object (e.g., furniture) with both hands without leaning on it. The body does not touch the stable object, and the legs support most of the body weight. Child thus stands with assistance for at least 10 seconds.

[i]Bayley gross motor subtest item #35: Child raises self to standing position using chair or other convenient object for support.

[j]Bayley gross motor subtest item #40: Stands alone. Child stands alone for at least 3 seconds after you release his or her hands.

[k]WHO-MGRS definition: Standing alone. Child stands in upright position on both feet (not on the toes) with the back straight. The legs support 100% of the child's weight. There is no contact with a person or object. Child stands alone for at least 10 seconds.
[l]Bayley gross motor subtest item #37: Walks with assistance. Child walks by making coordinated, alternated stepping movements.
[m]WHO-MGRS definition: Walking with assistance. Child is in upright position with the back straight. Child makes sideways or forward steps by holding onto a stable object (e.g., furniture) with 1 or both hands. One leg moves forward while the other supports part of the body weight. Child takes at least 5 steps in this manner.
[n]Bayley gross motor subtest item #43: Walks alone. Child takes at least 5 steps independently, displaying coordination and balance.
[o]WHO-MGRS definition: Walking alone. Child takes at least 5 steps independently in upright position with the back straight. One leg moves forward while the other supports most of the body weight. There is no contact with a person or object.
[p]"At screening" milestone presented at baseline was not video confirmed.
Note: Although the demonstration of the Stands with Assistance (Bayley gross motor subtest item #33) milestone for Patient 11 was captured and confirmed via a home video which was recorded prior to the patient's Visit 10-Month 9 clinic visit, the milestone achievement date is reflective of the date of the Visit 10-Month 9 clinic visit.
[q]Patient 9 experienced an infusion interruption during administration of onasemnogene abeparvovec. The drug infusion was delayed due to a pump malfunction. After the line was flushed, the patient received the entire dose of onasemnogene abeparvovec.

Supplemental Table 3. Bayley raw and scaled scores at all time points

		Observed values			Change from baseline values			
**Motor scale**	**Visit**	***n***	**Mean (SD)**	**Median (range)**	***n***	**BL mean**	**Mean (SD)**	**Median (range)**
**Fine motor (raw)**	Baseline	14	3.1 (1.69)	3.0 (0–6)				
	Day 30/Month 1	14	4.4 (2.41)	4.0 (1–8)	13	3.1	1.6 (1.94)	2.0 (–2 to 5)
	Month 2	4	6.0 (0.82)	6.0 (5–7)	4	1.8	4.3 (1.50)	4.0 (3–6)
	Age 3 months	14	8.2 (1.58)	9.0 (6–11)	13	3.0	5.3 (2.56)	6.0 (1–10)
	Age 6 months	14	18.4 (4.05)	19.0 (10–23)	13	2.9	15.6 (3.84)	16.0 (7–21)
	Age 9 months	14	23.9 (1.99)	24.5 (20–26)	13	3.1	20.6 (2.40)	20.0 (17–25)
	Age 12 months	10	28.5 (1.96)	28.5 (24–31)	9	2.8	25.7 (2.74)	26.0 (19–28)
	Age 15 months	8	33.0 (1.31)	33.0 (31–35)	8	2.9	30.1 (2.10)	31.0 (26–32)
	Age 18 months	9	34.2 (2.05)	34.0 (31–38)	8	3.1	30.6 (2.26)	30.5 (28–35)
	Age 21 months	11	37.8 (2.52)	39.0 (33–41)	10	3.0	34.7 (2.26)	34.0 (32–38)
	Age 24 months	10	40.1 (2.28)	40.5 (36–43)	10	3.1	37.0 (2.05)	36.5 (33–40)
**Fine motor (scaled)**	Baseline	14	9.6 (3.39)	9.0 (5–19)				
	Day 30/Month 1	14	9.7 (2.64)	9.5 (5–13)	13	9.6	0.4 (3.91)	2.0 (–10 to 4)
	Month 2	4	11.0 (0.82)	11.0 (10–12)	4	7.3	3.8 (2.06)	3.5 (2–6)
	Age 3 months	14	11.0 (1.71)	11.0 (8–14)	13	9.5	1.5 (3.62)	1.0 (–6 to 7)
	Age 6 months	14	9.1 (4.39)	10.0 (1–14)	13	9.5	–0.2 (4.64)	0.0 (–8 to 7)
	Age 9 months	14	8.9 (1.99)	9.5 (5–11)	13	9.6	–0.9 (4.42)	–1.0 (–13 to 5)
	Age 12 months	10	9.6 (1.78)	9.5 (6–13)	9	9.6	0.0 (4.69)	1.0 (–10 to 4)
	Age 15 months	8	12.0 (1.31)	12.0 (10–14)	8	9.8	2.3 (3.73)	3.5 (–5 to 6)
	Age 18 months	9	10.2 (2.05)	10.0 (7–14)	8	8.9	0.9 (2.59)	0.5 (–2 to 6)
	Age 21 months	11	11.5 (2.34)	12.0 (7–15)	10	8.7	2.7 (2.45)	2.0 (0–7)
	Age 24 months	10	11.4 (1.90)	11.5 (8–14)	10	9.9	1.5 (3.27)	1.5 (–6 to 5)
**Gross motor (raw)**	Baseline	14	6.0 (2.91)	5.0 (2–12)				
	Day 30/Month 1	14	9.1 (3.28)	8.5 (4–15)	13	6.1	2.6 (2.26)	2.0 (–2 to 7)
	Month 2	4	10.8 (1.89)	11.5 (8–12)	4	4.3	6.5 (3.00)	6.0 (4–10)
	Age 3 months	14	13.9 (2.32)	14.0 (9–18)	13	5.8	7.7 (3.28)	9.0 (2–12)
	Age 6 months	14	22.7 (3.15)	22.0 (17–28)	13	5.9	16.6 (4.25)	17.0 (11–24)
	Age 9 months	14	31.8 (3.24)	32.5 (27–37)	13	6.1	26.1 (4.11)	25.0 (21–32)
	Age 12 months	10	39.9 (3.14)	40.5 (33–44)	9	6.4	33.6 (5.25)	34.0 (26–41)
	Age 15 months	8	45.5 (1.77)	45.5 (43–49)	8	6.6	38.9 (2.36)	38.5 (36–43)
	Age 18 months	9	48.8 (2.99)	49.0 (44–52)	8	5.3	43.1 (3.87)	43.5 (37–48)
	Age 21 months	11	51.3 (3.04)	51.0 (46–57)	10	6.2	44.9 (2.56)	45.5 (41–50)

		Observed values			Change from baseline values			
**Motor scale**	**Visit**	***n***	**Mean (SD)**	**Median (range)**	***n***	**BL mean**	**Mean (SD)**	**Median (range)**
	Age 24 months	10	53.8 (3.43)	55.0 (47–59)	10	5.5	48.3 (3.13)	47.0 (45–54)
**Gross motor (scaled)**	Baseline	14	10.3 (1.98)	10.0 (7–14)				
	Day 30/Month 1	14	11.0 (2.29)	11.0 (8–15)	13	10.3	0.4 (1.98)	1.0 (–5 to 3)
	Month 2	4	11.3 (1.50)	12.0 (9–12)	4	9.0	2.3 (2.63)	2.0 (0–5)
	Age 3 months	14	11.8 (1.53)	11.5 (9–15)	13	10.2	1.4 (2.22)	1.0 (–3 to 5)
	Age 6 months	14	8.9 (2.43)	8.0 (5–13)	13	10.2	–1.5 (3.26)	–1.0 (–6 to 4)
	Age 9 months	14	7.9 (2.20)	8.5 (5–12)	13	10.3	–2.2 (2.79)	–3.0 (–6 to 2)
	Age 12 months	10	8.6 (2.41)	9.0 (3–12)	9	10.4	–1.8 (3.93)	–2.0 (–8 to 4)
	Age 15 months	8	8.9 (1.13)	9.0 (7–11)	8	10.6	–1.8 (1.91)	–1.5 (–5 to 1)
	Age 18 months	9	8.6 (1.67)	9.0 (6–10)	8	9.9	–1.5 (2.78)	–1.0 (–6 to 2)
	Age 21 months	11	8.5 (1.81)	9.0 (5–12)	10	10.3	–1.9 (1.91)	–1.5 (–5 to 1)
	Age 24 months	10	8.5 (2.07)	9.0 (5–12)	10	10.1	–1.6 (2.12)	–2.5 (–4 to 2)

BL, baseline; SD, standard deviation.

Note: *n* = 6 males and *n* = 9 females; mean (SD) age at dosing, 28.7 (11.68) days.

Supplemental Table 4. Percentage of children achieving a scaled score on Bayley gross and fine motor subtests ≥4 (±2 SD) up to 24 months of age

**Percentage achieving scaled score ≥4 on BSID gross and fine motor subtests**<br>**Visit**	***n*/*N* (%)[a]**
At any point post-baseline	15/15 (100)
At all post-baseline visits	0/2 (0)
At Day 30/Month 1	14/14 (100)
At Month 2	4/4 (100.0)
At age 3 months	14/14 (100.0)
At age 6 months	11/14 (79)
At age 9 months	14/14 (100)
At age 12 months	9/10 (90)
At age 15 months	8/8 (100)
At age 18 months	9/9 (100)
At age 21 months	11/11 (100)
At age 24 months	10/10 (100)

BSID, Bayley Scales of Infant and Toddler Development; ITT, intention-to-treat; SD, standard deviation.

[a]ITT population, *n* = 6 males and *n* = 9 females; mean (SD) age at dosing, 28.7 (11.68) days.

Supplemental Table 5. Summary of changes from baseline to maximum post-baseline values in CMAP (ITT population)

**CMAP parameter**<br>**Visit**	***n*[a]**	**Mean (SD)**	**Median (range)**	**LS mean (SE)**	***P*-value**
**CMAP amplitude (mV)[b]**					
Baseline	15	4.26 (1.075)	4.10 (2.7–7.0)		
Maximum post-baseline value	15	6.18 (1.252)	6.00 (4.2–8.5)		
Change from baseline	15	1.92 (1.779)	1.80 (–0.6 to 5.0)	1.92 (0.331)	<0.0001
**Mixed model with repeated measurements analysis[c]**					
Month 6 – observed	13	5.28 (1.381)	5.20 (2.6–7.6)		
Month 6 – change from baseline	13	1.15 (1.965)	0.80 (–2.2 to 4.2)	0.94 (0.351)	0.0168
Month 12 – observed	9	4.81 (0.833)	4.90 (3.4–5.8)		
Month 12 – change from baseline	9	0.56 (1.033)	0.80 (–1.2 to 1.8)	0.75 (0.399)	0.0782
Month 18 – observed	7	5.96 (1.471)	5.70 (4.2–8.5)		
Month 18 – change from baseline	7	1.80 (1.860)	1.90 (–0.2 to 5.0)	1.73 (0.443)	0.0013
Month 24 – observed	8	5.59 (1.316)	5.40 (3.6–7.8)		
Month 24 – change from baseline	8	1.26 (1.193)	1.15 (0.0–2.7)	1.57 (0.419)	0.0018

CMAP, compound muscle action potential; ITT, intention-to-treat; LS, least squares; SE, standard error.

[a]ITT population, *n* = 6 males and *n* = 9 females; mean (SD) age at dosing, 28.7 (11.68) days.

[b]An analysis of covariance model was used for the analysis with maximum change from baseline in CMAP as the dependent variable and baseline value as a covariate. The maximum value for each CMAP parameter was the maximum value observed at any time post-baseline, including unscheduled and out of window assessments.

[c]The statistical model is a mixed model with repeated measurements with change from baseline in CMAP as the dependent variable, fixed effect of visit, a covariate of baseline value, age at baseline (days), and interaction of baseline and visit. A compound symmetry covariance structure was used to model the within-patients errors. Only scheduled visits (Month 6, Month 12, Month 18, and Month 24) are included in the model.

Supplemental Table 6. Treatment-emergent adverse events observed for two or more children

**Preferred term**	***N* = 15[a]** ***n* (%)**
Any TEAE	15 (100)
Pyrexia	11 (73)
Upper respiratory tract infection	9 (60)
Teething	5 (33)
Aspartate aminotransferase increased	4 (27)
Cough	4 (27)
Diarrhea	4 (27)
Dermatitis diaper	3 (20)
Alanine aminotransferase increased	3 (20)
Gastroesophageal reflux disease	3 (20)
Nasopharyngitis	3 (20)
Otitis media	3 (20)
Blood calcium increased	2 (13)
Blood creatinine phosphate MB increased	2 (13)
Microcytic anemia	2 (13)
Gastroenteritis	2 (13)
Hand-foot-and-mouth disease	2 (13)
Hypotonia	2 (13)
Nasal congestion	2 (13)
Rash	2 (13)
Troponin increased	2 (13)
Urinary tract infection	2 (13)
Vomiting	2 (13)

TEAE, treatment-emergent adverse event.

[a]Safety population, *n* = 6 males and *n* = 9 females; mean (SD) age at dosing, 28.7 (11.68) days.

Supplemental Table 7. Treatment-related adverse events by system organ class

**System organ class**<br>**Preferred term**	***N* = 15[a]**<br>***n* (%)**
Any TEAE	8 (53)
Investigations	7 (47)
Aspartate aminotransferase increased	4 (27)
Alanine aminotransferase increased	3 (20)
Blood creatine phosphokinase MB increased	2 (13)
Troponin increased	2 (13)
Blood alkaline phosphatase increased	1 (7)
Gamma-glutamyltransferase increased	1 (7)
Platelet count increased	1 (7)
Metabolism and nutrition disorders	2 (13)
Poor feeding infant	1 (7)
Weight gain poor	1 (7)
Psychiatric disorders	1 (7)
Agitation	1 (7)
Respiratory, thoracic, and mediastinal disorders	1 (7)
Cough	1 (7)
Gastrointestinal disorders	5 (36)
Gastroesophageal reflux disease	2 (13)
Vomiting	1 (7)
Skin and subcutaneous system disorders	3 (20)
Dermatitis diaper	1 (7)
Lipohypertrophy	1 (7)
Pruritis	1 (7)
Rash macular	1 (7)
Rash	1 (7)
Blood and lymphatic system disorders	1 (7)
Iron deficiency anemia	1 (7)
Endocrine disorders	1 (7)
Cushingoid	1 (7)
General disorders and administration site conditions	1 (7)
Pyrexia	1 (7)
Infections and infestations	1 (7)
Nasopharyngitis	1 (7)

TEAE, treatment-emergent adverse event.
[a]Safety population, *n* = 6 males and *n* = 9 females; mean (SD) age at dosing, 28.7 (11.68) days.

Supplemental Table 8. Hepatotoxicity-related treatment-emergent adverse events of special interest

Patient/ Age (days)[a] /Weight (kg)[b] at baseline	Preferred term	CTCAE grade	Start/end (study day)	Outcome	Causality	Prednisolone/prednisolone equivalent dose
Patient 7/ 37/3.8	Alanine aminotransferase increased	Grade 2	42/147	Recovered/resolved	Definitely related	1 mg/kg Days –1 to 29<br>1.25 mg/kg Days 30 to 36<br>1.5 mg/kg Days 37 to 42<br><br>Patient continued to receive prednisolone, with doses ranging from 1 to 1.8 mg/kg Days 43 to 165 and doses below 1 mg/kg from Days 166 to 320<br><br>Total days = 321
	Aspartate aminotransferase increased	Grade 2	42/147	Recovered/resolved	Definitely related	
Patient 9/ 21/4.2	Aspartate aminotransferase increased	Grade 2	42/49	Recovered/resolved	Probably related	1 mg/kg Days –1 to 53<br>1.2 mg/kg Days 54 to 76<br>0.6 mg/kg Days 77 to 88<br>0.3 mg/kg Days 89 to 92<br>0.15 mg/kg Days 93 to 114<br><br>Total days = 115
		Grade 1	49/69	Recovered/resolved	Probably related	
	Alanine aminotransferase increased	Grade 3	42/56	Recovered/resolved	Probably related	
		Grade 2	56/69	Recovered/resolved	Probably related	
		Grade 1	69/172	Recovered/resolved	Probably related	
Patient 10/ 36/4.3	Aspartate aminotransferase increased	Grade 1	7/14	Recovered/resolved	Probably related	1 mg/kg Days –1 to 31<br>0.5 mg/kg Days 32 to 45<br>0.25 mg/kg Days 46 to 59<br>1 mg/kg Days 74 to 82<br>0.75 mg/kg Days 82 to 95<br>0.5 mg/kg Days 95 to 106<br>0.25 mg/day Days 106 to 124
	Gamma glutamyltransferase increased	Grade 1	7/14	Recovered/resolved	Probably related	
	Aspartate aminotransferase increased	Grade 2	70/140	Recovered/resolved	Probably related	

Patient/ Age (days)[a] /Weight (kg)[b] at baseline	Preferred term	CTCAE grade	Start/end (study day)	Outcome	Causality	Prednisolone/prednisolone equivalent dose
						Total days = 114
Patient 11/ 9/3.9	Alanine aminotransferase increased	Grade 1	32/63	Recovered/resolved	Probably related	1 mg/kg Days –1 to 32<br>0.5 mg/kg Days 33 to 34<br>1 mg/kg Days 35 to 69
	Aspartate aminotransferase increased	Grade 1	32/63	Recovered/resolved	Probably related	0.5 mg/kg Days 70 to 84<br>0.25 mg/kg Days 85 to 98<br><br>Total days = 99

CTCAE, Common Terminology Criteria for Adverse Events.

[a]Age at dosing (dose date − date of birth + 1).

[b]Weight at baseline.

Supplemental Table 9. Thrombocytopenia-related treatment-emergent adverse events of special interest

Patient/ Age (days)[a] /Weight (kg)[b] at baseline	Preferred term	CTCAE grade	Start/end (study day)	Platelets ($\times 10^9$/L)[3]	Outcome	Causality
Patient 1/ 29/4.7	Contusion	Grade 1	9/18	Day 9: 333<br>Day 23: 925<br>Normal 130–400	Recovered/resolved	Unrelated
Patient 7/ 37/3.8	Hematemesis	Grade 2	26/26	Day 21: 424<br>Day 35: 420<br>Day 42: 430	Recovered/resolved	Unrelated
	Hematochezia	Grade 1	45/45	Day 49: 478<br>Normal: 130–400	Recovered/resolved	Unrelated

CTCAE, Common Terminology Criteria for Adverse Events.

[a]Age at dosing (dose date − date of birth + 1).

[b]Weight at baseline.

Supplemental Table 10. Cardiac treatment-emergent adverse events of special interest

Patient Age[a] (days) /Weight (kg)[b]	Preferred term	CTCAE grade	Start/end (study day)	Outcome	Causality
Patient 2/ 39/4.4	Troponin increased	Grade 1	30/35	Recovered/resolved	Possibly related
Patient 4/ 9/3.9	CK-MB increased	Grade 1	8/32	Recovered/resolved	Probably related
	Troponin increased	Grade 1	13/21	Recovered/resolved	Probably related
Patient 6/ 18/3.6	CK-MB increased	Grade 1	440/–	Not recovered/not resolved	Possibly related

CTCAE, Common Terminology Criteria for Adverse Events.

[a]Age at dosing (dose date − date of birth + 1).

[b]Weight at baseline.

Supplemental Table 11. Sensory abnormalities suggestive of ganglionitis adverse events of special interest

Patient Age (days) /Weight (kg) at baseline	Preferred term	CTCAE grade	Start/end (study day)	Outcome	Causality	Prednisolone dose (mg/kg)
Patient 4/ 9/3.9	Areflexia	Grade 1	545/628	Recovered/resolved	Unrelated	Day –1 to 32: 1<br>Day 33–34: 0.5<br>Day 35–69: 1<br>Day 70–84: 0.5<br>Day 85–98: 0.25
	Areflexia	Grade 1	714/–	Not recovered/not resolved	Unrelated	

CTCAE, Common Terminology Criteria for Adverse Events.

Supplemental Table 12. Summary of identification of adverse events of special interest

- Hepatotoxicity, identified via the following SMQ:
  - Hepatic disorders (SMQ)
- Thrombocytopenia, identified via the following CMQ:
  - Transient thrombocytopenia (CMQ)
- Cardiac events, identified via the following SMQs:
  - Ischemic heart disease (SMQ)
  - Cardiomyopathy (SMQ)
  - Cardiac arrhythmias (SMQ)
  - Embolic and thrombotic events (SMQ)
  - Myocardial infarction (SMQ)
- Thrombotic microangiopathy, identified via the following approach:
  - Criteria #1: cases with any one of the following PTs: thrombotic microangiopathy OR hemolytic uremic syndrome OR atypical hemolytic uremic syndrome
  - Criteria #2: cases with at least one PT from each of the following SMQs representing thrombocytopenia, hemolysis and relevant renal events respectively:
    - Hematopoietic thrombocytopenia (SMQ)
    - Hemolytic disorders (SMQ)
    - Acute renal failure (SMQ) or renovascular disorders (SMQ)
- Sensory abnormalities suggestive of ganglionitis, identified via the following CMQ:
  - DRG cell inflammation CMQ

CMQ, Customized Medical Dictionary for Regulatory Activities query; PT, preferred term; SMQ, Standardized Medical Dictionary for Regulatory Activities query.
